# A decade after the metabolomics standards initiative it's time for a revision

**DOI:** 10.1038/sdata.2017.138

**Published:** 2017-09-26

**Authors:** Rachel A. Spicer, Reza Salek, Christoph Steinbeck

**Affiliations:** 1 European Bioinformatics Institute (EMBL-EBI), Hinxton, Cambridge CB10 1SD, UK; 2 Friedrich-Schiller-University, Fürstengraben 1, Jena 07743, Germany

**Keywords:** Research data, Metabolomics, Standards

## Comment

A recent analysis of publicly available metabolomics data shows that the MSI guidelines are not well abided to in publicly shared metabolomics studies. We propose that the MSI guidelines should be revisited and revised, as has been done in other communities, to fit the current community needs.

In the year 2005, leading experts in metabolomics, with support from the Metabolomics Society, gathered to discuss metabolomics experimental standards and formed the metabolomics standards initiative (MSI)^1^. Two years later, the MSI published a series of reporting standards for metabolomics^2–8^. Another 5 years passed until a network of global repositories^9–11^ for metabolomics data arose, to which the MSI guidelines could be applied. Now, as of 2nd August 2017, there are more than 700 metabolomics datasets publicly available via MetabolomeXchange (http://www.metabolomexchange.org/), which, for the first time, allows for an assessment of metabolomics data sharing and compliance with the MSI guidelines.

**Figure 1:**
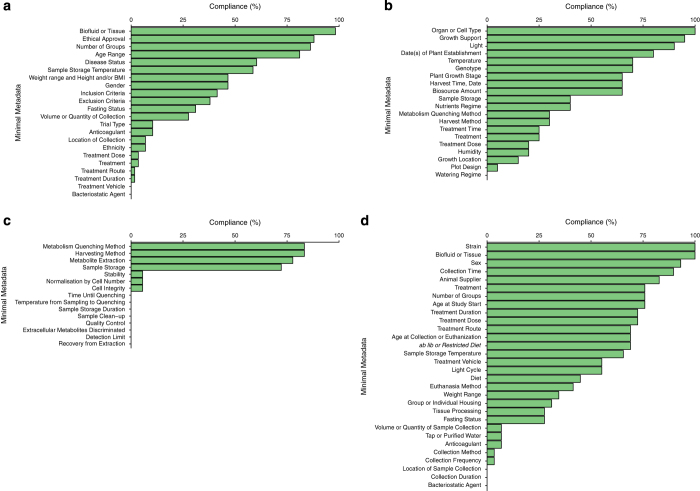
A decade after the metabolomics standards initiative it's time for a revision. The percentage of studies in MetaboLights that comply with the MSI minimal reporting standards. (**a**) Clinical *Homo sapiens* studies compliance with the mammalian clinical trials and human studies minimal reporting standards. (**b**) *Arabidopsis thaliana* studies compliance with the plant minimal reporting standards. (**c**) *In vitro H. sapiens* studies using human cells and cell lines compliance with the microbial and *in vitro* minimal reporting standards. (**d**) Pre-clinical *Mus musculus* studies compliance with the pre-clinical minimal reporting standards. The percentage compliance with the MSI reporting standards is similar for all repositories (see Spicer *et al.*^14^).

In the decade since the first publication of the MSI reporting standards, the metabolomics community has matured. There is now greater understanding of genetic and environmental factors that can induce significant effects on the metabolome. The general purpose metabolomics data repositories MetaboLights^9^ and Metabolomics Workbench^10^, along with the smaller more specific repositories, MetaPhen^12^ and MeRy-B^13^, were designed to adhere with the MSI guidelines for minimum metadata reporting. We have performed a study assessing the compliance of the datasets in the MetaboLights, Metabolomics Workbench, MetaPhen, and MeRy-B repositories. It was found that many of the MSI’s minimal reporting standards are not complied with by many of the studies in these repositories (Fig. 1 shows examples from the MetaboLights repository). There were no MSI standards that were complied with in every publicly available study. There are, however, minimal reporting standards that are not applicable to every study e.g., treatment, which is only relevant to studies including a treatment. Being only applicable to a small subset of studies may result in the low percentage compliance to these standards (Fig. 1).

While all databases under investigation claim to demand compliance with the MSI guidelines, the true compliance is unexpectedly low. We have detailed our finding in Spicer *et al.*^14^ and will comment only on the main points below.

The current MSI reporting standards suffer from a variety of limitations. They are difficult to interpret and do not capture all metadata required for re- and further data analysis. Between the biological context metadata subgroups there is also a lack of consistency as to what constitutes a minimal and best practice reporting standard. Additionally there is unnecessary repetition of standards between the biological context metadata and the chemical analysis working group (CAWG) guidelines^3^.

Compliance with minimum information standards and related database submission procedures has always been a source for despair. Unless experiments and technological setup are carefully planned to take into account later data and metadata submission procedure, the experience of converting lab book entries into an ISA^15^ compliant dataset ready and acceptable for deposition is cumbersome, to say the least. Nevertheless, one would assume that the guidance of groups of world-leading experts in metabolomics in the form of minimum information (MI) standards would be enthusiastically welcomed and used. This is not the case. We believe that a major reason for this is the sheer inconvenience of recording rich meta-data, which might not seem relevant for one’s own study at a given point in time. Furthermore, repositories do not agree on the set of metadata required and, in particular during their maturation stage, do not vigorously enforce MI standard compliance in fear of annoying submitters.

There have been efforts by the Metabolite Identification Task Group of the Metabolomics Society to reassess the reporting standards for metabolite identification^16^ and the community has suggested several improvements^17–19^. The recently launched MEtabolomics standaRds Initiative in Toxicology (MERIT) (http://www.ecetoc.org/topics/standardisation-metabolomics-assays-regulatory-toxicology/) will define best practice and minimal reporting standards for the application of metabolomics to regulatory toxicology. However, there have not been advances in the reporting standards for other areas of metabolomics. We believe that now, after putting the MSI biological context guidelines to test in ~400 publically available datasets, the MSI guidelines should be revisited and revised, and dataset submission procedures in MetabolomeXchange should be harmonized. The community, along with data curators, publishers and funders should be consulted. Consideration should be given to the minimum amount of metadata required to be able to a) repeat an experiment and b) re-analyse the data. The data that are now publically available in a number of open-access repositories around the globe will be a treasure trove for guiding the selection of minimal reporting standards.

## Additional information

**How to cite this article**: Spicer, R. A. *et al.* A decade after the metabolomics standards initiative it's time for a revision. *Sci. Data* 4:170138 doi: 10.1038/sdata.2017.138 (2017).
